# Climate‐related range shifts in Arctic‐breeding shorebirds

**DOI:** 10.1002/ece3.9797

**Published:** 2023-02-07

**Authors:** Christine M. Anderson, Lenore Fahrig, Jennie Rausch, Jean‐Louis Martin, Tanguy Daufresne, Paul A. Smith

**Affiliations:** ^1^ Department of Biology, Geomatics and Landscape Ecology Laboratory Carleton University Ottawa Ontario Canada; ^2^ Canadian Wildlife Service Environment and Climate Change Canada Yellowknife Northwest Territories Canada; ^3^ Centre d'Écologie Fonctionnelle et Évolutive CNRS Montpellier Cedex 5 France; ^4^ UMR Eco&Sols INRAE Montpellier Cedex 2 France; ^5^ Wildlife Research Division Environment and Climate Change Canada Ottawa Ontario Canada

**Keywords:** climate tracking, global change, poleward shifts, range dynamics, Re‐distribution, wader

## Abstract

**Aim:**

To test whether the occupancy of shorebirds has changed in the eastern Canadian Arctic, and whether these changes could indicate that shorebird distributions are shifting in response to long‐term climate change.

**Location:**

Foxe Basin and Rasmussen Lowlands, Nunavut, Canada.

**Methods:**

We used a unique set of observations, made 25 years apart, using general linear models to test if there was a relationship between changes in shorebird species' occupancy and their species temperature Index, a simple version of a species climate envelope.

**Results:**

Changes in occupancy and density varied widely across species, with some increasing and some decreasing. This is despite that overall population trends are known to be negative for all of these species based on surveys during migration. The changes in occupancy that we observed were positively related to the species temperature index, such that the warmer‐breeding species appear to be moving into these regions, while colder‐breeding species appear to be shifting out of the regions, likely northward.

**Main Conclusions:**

Our results suggest that we should be concerned about declining breeding habitat availability for bird species whose current breeding ranges are centered on higher and colder latitudes.

## Graphical abstract

We used a unique set of observations, made 25 years apart, using general linear models to test if there was a relationship between changes in shorebird species' occupancy and their species temperature index, a simple version of a species' climate envelope. The changes in occupancy that we observed were positively related to the species temperature index, such that the warmer‐breeding species appear to be moving into these regions, while colder‐breeding species appear to be shifting out of the regions, likely northward. Our results suggest that we should be concerned about declining breeding habitat availability for bird species whose current breeding ranges are centered on higher and colder latitudes.

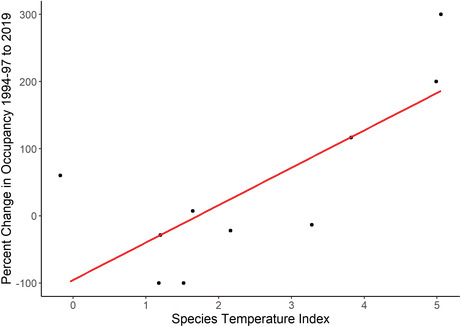

## 1 INTRODUCTION

Over the past century, many species have shifted their distributions in response to anthropogenic influences. One increasingly important driver of distributional shifts is climate change, with species moving toward higher latitudes and higher elevations in response to a warming climate (Chen et al., 2011; Parmesan & Yohe, 2003). For example, the northern limit of birds' ranges measured by the North American Breeding Bird Survey shifted northward at a rate of 2.35 km/year between 1967 and 2002 (Hitch & Leberg, 2007). Likewise, butterfly and moth ranges have expanded northward in Finland (Mikkola, 1997), Great Britain (Hill et al., 2002), and across Europe (Parmesan et al., 1999). These changes in species' range limits are an important measure of how species are redistributing in response to climate change. Patterns of species density and community composition are shifting as well, creating novel ecological communities (Devictor et al., 2012; Kampichler et al., 2012; Lurgi et al., 2012). Estimating the distribution of species has become a very active field of research, responding to concerns about how accelerating global environmental change will reshape the world's ecosystems (Guisan & Thuiller, 2005).

Identifying shifts in distribution in response to climate change requires long‐term and large‐scale species data. However, the regions where the climate is changing fastest are often those where such data are sparse, making it challenging to measure shifts in species distribution (Daskalova et al., 2023; Shirey et al., 2021). For example, temperatures in the Arctic are rising three times faster than the global average (AMAP, 2021). However, relative to other terrestrial biomes, consistent data collection efforts focused on describing species abundance and distributions are limited in the Arctic (Aronsson et al., 2021; Smith et al., 2020) due to logistical constraints associated with conducting field work at high latitudes (Mallory et al., 2018). Furthermore, citizen science programs such as the Christmas Bird Count, eBird, and Breeding Bird Survey programs (Curley et al., 2020; Devictor et al., 2008; Johnston et al., 2021; Lindström et al., 2013) are not viable in the Arctic because the region is very sparsely inhabited. As such, regularly repeated, large‐scale surveys of vertebrate populations are currently lacking in the Arctic, which reduces the ability to assess patterns in species distributions as a function of shifts in climate conditions.

Despite the challenges, information about species distributions in the Arctic will be increasingly important for supporting conservation policies and protected areas being developed to protect northern species from increasing human presence and a rapidly warming climate. Melting ice is likely to lead to increases in shipping and resource extraction (Arbo et al., 2013). Arctic species are also particularly vulnerable to climate change due to three unique geographic factors that are leading to an “Arctic squeeze” which has the potential to dramatically limit the capacity of Arctic species to adaptively shift their ranges (Meltofte et al., 2007; Vincent, 2020). First, the surface area of the Earth decreases as latitude increases toward a fixed end point at the pole, limiting options for northern expansion of habitats (Gilg et al., 2012). Second, northern expansion of terrestrial habitats cannot occur in regions that are bordered to the north by the Arctic Ocean; in many locations, there is no more northerly landmass available for terrestrial species to expand into (Wauchope et al., 2017). Third, the southern border of tundra habitat is moving northward, as shrubs and trees also shift northward in response to climate change, encroaching into the open habitats preferred by many tundra‐breeding species (García Criado et al., 2020; Martin et al., 2017).

**FIGURE 1:**
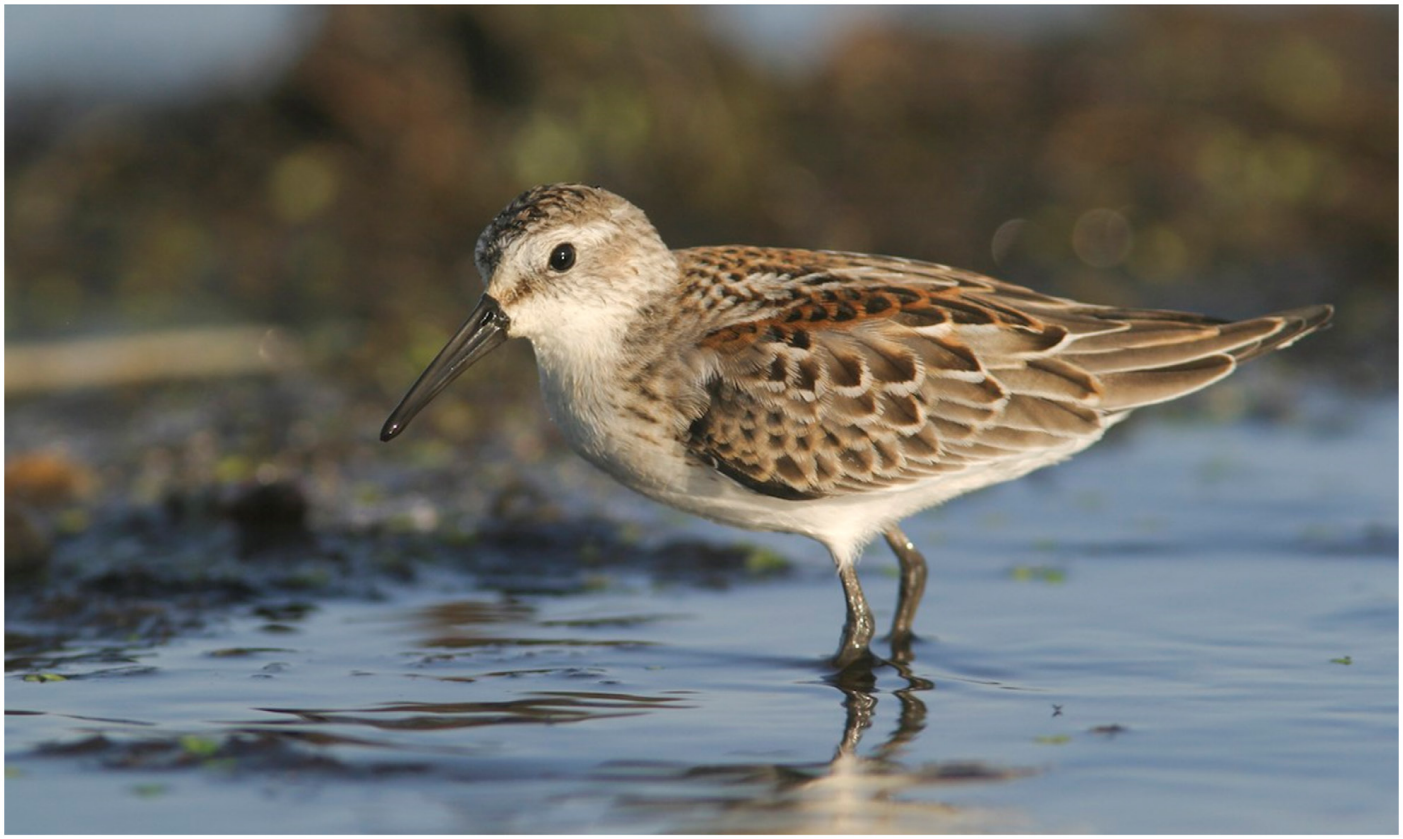
White‐rumped Sandpiper, 1 of the 12 species of shorebirds observed in this study. Shorebirds are the most abundant and diverse group of birds in many tundra habitats.

Here, we investigate whether the breeding distributions of shorebirds have shifted northward over a 25‐year period in the Canadian Arctic. We focus here on shorebirds, the most abundant and diverse group of birds in many tundra habitats (Figure 1; Smith et al., 2020). These species are likely to be particularly sensitive to climate change because of their highly migratory life history, as Arctic‐breeding shorebirds undertake long, energetically expensive migrations, only have a short window available for breeding in the Arctic, and depend on ecological synchronicities with their invertebrate prey (Galbraith et al., 2014; Kwon et al., 2019). Surveys providing an index of shorebird abundance during their migrations through southern Canada and the United States suggest that shorebird populations have experienced pronounced declines in the past 50 years, including all of the species studied here (Bart & Johnston, 2012; Smith et al., 2020, 2023). These declines are often attributed to habitat loss and degradation at migratory stopovers and non‐breeding sites (Thomas et al., 2006), but given that climate change in the Arctic is expected to be rapid and severe, there is concern that environmental changes to shorebird‐breeding habitats may increasingly cause additional stress in these declining populations (Galbraith et al., 2014).

**FIGURE 2:**
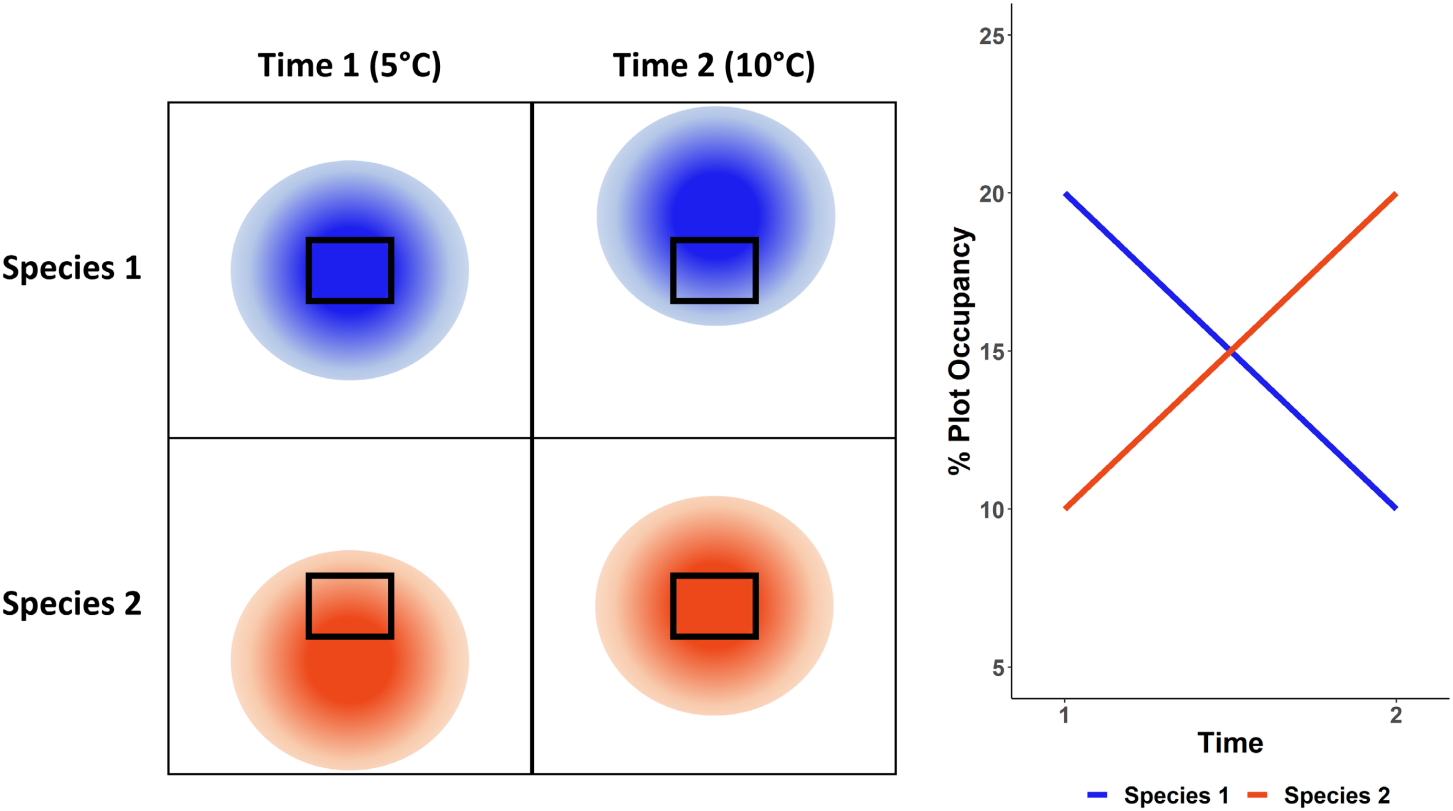
Expected changes in plot occupancy as species distributions shift north in response to warming temperatures. Species are assumed to have higher occupancy in the center of their range and lower occupancy at the edges of their range (Gaston, 2003; indicated in this figure by opacity of the orange and blue species ranges). Plots are surveyed within the study area outlined by the black box. The STI (mean June temperature of the range) for species 1 is 5°C; in this case, making it a colder‐breeding species. At time 1, the mean temperature of the study area is also 5°C, therefore, the occupancy of species 1 is high. At time 2, the mean temperature of the study area has increased to 10°C. Species 1 has shifted its distribution northward. The study area is now on the southern edge of its range, and the occupancy of species 1 has declined. The STI of species 2, a warmer‐breeding species, is 10°C. At time 1, the study area is at the northern edge of its range, therefore, the occupancy of species 2 at time 1 is low. At time 2, the study area is now in the center of its range and the occupancy of species 2 has increased.

We used a unique set of observations, made 25 years apart across 50,000 km^2^ of mid‐Arctic tundra habitats, to test whether the occupancy of shorebirds has changed over time, and whether these changes could indicate that distributions are shifting in response to long‐term climate change. These data were collected as part of the Arctic Program for Regional and International Shorebird Monitoring (PRISM), an unprecedented Arctic‐wide survey that will eventually track changes in the population size, trends, and distribution of shorebirds (Bart & Johnston, 2012). The observed summer temperature in northern Canada has increased by 1.6°C between 1948 and 2016 (Zhang et al., 2019). We therefore predicted that at mid‐Arctic latitudes, species associated with warmer low Arctic‐breeding habitats should be moving into the region and observed more frequently, and species associated with colder High Arctic habitats should be moving out of the region and observed less frequently (Jiguet et al., 2010) (Figure 2). To test this prediction, we represented species temperature associations using the species temperature index (STI), a simple version of a species’ climate envelope. Given that the population trends for these species are negative, we were interested to look at overall trends in survey counts in these regions to give context to any potential distribution shifts.

The STI is the long‐term average temperature experienced by individuals of a species across their breeding range (Devictor et al., 2008). While species distributions are much more complex than simple climate relationships, this index has been a useful approach for describing how population trends and demography of bird populations are responding to climate change (Gaüzère et al., 2020; Godet et al., 2011; Princé & Zuckerberg, 2015). The collective contributions of individual species responses can give an indication of how the ecological community is responding to change (Curley et al., 2022; Gaüzère et al., 2019). Species with low STI consistently show more negative population trends in response to high temperatures (Pearce‐Higgins et al., 2015). We thus predicted a positive relationship between STI and the temporal change in shorebird occupancy, with occupancy increasing for warmer‐breeding species and occupancy decreasing for colder‐breeding species over 25 years. Given that the large‐scale population trends for these species are negative (Bart & Johnston, 2012; Smith et al., 2020, 2023), for any species that have positive regional trends, this can be interpreted as reflecting distributional change, rather than change in population‐level abundance.

## 2 METHODS

### 2.1 Shorebird surveys

**FIGURE 3:**
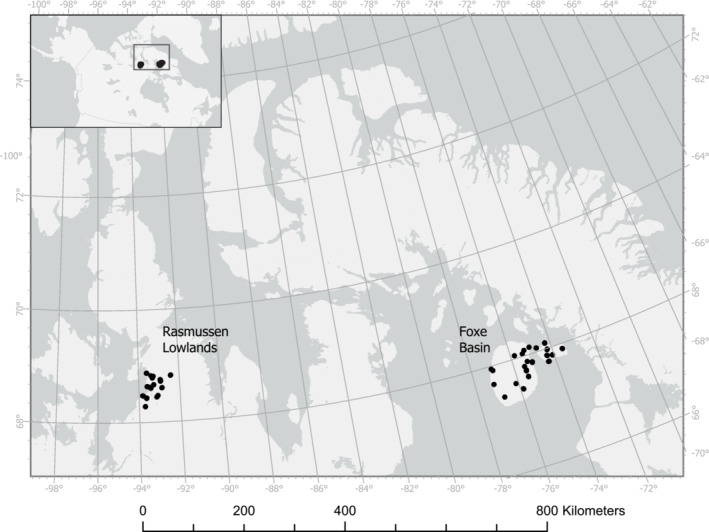
Map of Eastern Arctic study areas showing plots where breeding shorebirds were surveyed in 1994–1997 and then 22 to 25 years later in 2019.

Surveys were conducted as part of the Arctic PRISM shorebird monitoring program (Bart & Johnston, 2012). These surveys were designed to estimate population sizes and habitat relationships for shorebirds breeding across the whole of the North American Arctic, only recently completing the first round of surveys that covers each of the 19 PRISM survey regions. Here, we include data from the first two regions to be surveyed for a second time in the Canadian Arctic. We surveyed 25,000 km^2^ in the Rasmussen lowlands, a coastal plain wetland complex located at the base of the Boothia Peninsula, designated as a Ramsar Wetland of International Importance (Carp, 1980) and a Canadian Important Bird Area (Aguilar Mugica et al., 2009). We also surveyed a 25,000 km^2^ area in Foxe Basin including the coastal wetlands and inland dry areas on Prince Charles Island, several nearby islands, and a portion of western Baffin Island (Figure 3). Both regions, approximately 750 km apart, include a wide variety of land cover types, such as intertidal flats, low‐lying salt marshes, flat marshy tundra, heath tundra, dry grasslands, beach ridge complexes, and unvegetated broken shale (Bart & Johnston, 2012). Each region was stratified by habitat type, and plots were randomly sampled within each habitat type, with a greater proportion of plots located in wetland habitats (Bart & Johnston, 2012). These two regions are well suited for the current study because they are high‐quality shorebird habitat, containing a good diversity and abundance of shorebirds, and because they are located at mid‐Arctic latitudes, therefore hosting both colder‐ and warmer‐breeding species.

Each region was surveyed twice, 22–25 years apart. The Rasmussen lowlands region was surveyed in 1994–1995 and 2019, while the Foxe Basin region was surveyed in 1996–1997 and 2019. While the earlier surveys of each region were completed over sequential 2‐year periods, the later surveys in both regions were completed simultaneously in one season owing to increased funding and resources. We excluded the 1996 data because of unusually poor weather and flooding that disrupted the normal behavior of the birds (Bart & Johnston, 2012). Each plot was surveyed once per survey period (1994–1997 and 2019). Surveys were conducted between June 18 and July 15, during late courtship and early incubation, when breeding territories could be identified from the birds' territorial displays. The detectability of birds is believed to change throughout the season, as birds settle on their nests and incubation progresses. The late courtship to early incubation period maximizes detectability. Detectability could vary slightly even within this time window, and most certainly varies due to weather and other factors. This variability in detection could introduce imprecision into the estimates. However, we do not expect that this would contribute to bias because surveys in both time periods occurred over a number of days during late courtship–early incubation. In both time periods, 28 plots were surveyed in the Rasmussen Lowlands and 36 plots were surveyed in Foxe Basin (64 plots total). Plots were 16 ha (400 by 400 m). Surveyors recorded the proportion of each plot covered by upland habitat (e.g., mesic grasslands or heath habitats, and sparsely vegetated xeric habitats) or lowland habitat (e.g., hydric areas of grass/sedge, polygonal wetlands, and saltmarsh). In our analyses, we categorized a plot as upland or lowland depending on which habitat type was predominant.

Following PRISM protocols (Bart & Johnston, 2012), surveyors searched the plot walking straight‐line transects, covering a breadth of 50 m with the observers situated 25 m apart, using a GPS to ensure complete coverage of the entire plot. Surveys took approximately 90 mins. Observers recorded the number and species of all birds observed within each plot. Altogether we observed 12 species of shorebirds: American Golden Plover (*Pluvialus dominica*), Baird's Sandpiper (*Calidris bairdii*), Black‐bellied Plover (*Pluvialis squatrola*), Buff‐breasted Sandpiper (*Tryngites subruficollis*), Dunlin (*Calidris alpina*), Pectoral Sandpiper (*Calidris melanotos*), Red Knot (*Calidris cantus*), Red Phalarope (*Phalaropus fulicarius*), Ruddy Turnstone (*Arenaria interpres*), Semipalmated Sandpiper (*Calidris pusilla*), Stilt Sandpiper (*Calidris himantopus*), and White‐rumped Sandpiper (*Calidris fuscicollis*).

### 2.2 Analyses

We tested whether the occupancy of breeding shorebirds, as well as species richness and density of shorebirds changed over time in a generalized linear model (GLM) framework. We used logistic models for occupancy, and log‐linear models for species richness and density, using a negative binomial distribution to account for extra zeros in the count data. We considered a structurally identical model for each response variable. Our inferential model consisted of an additive categorical effect of time period (i.e., 1994–1997 or 2019), which served as our index of temporal shifts in species distribution. Likewise, we considered additive categorical effects of region (i.e., Rasmussen or Foxe Basin) and habitat type (i.e., upland or lowland) to account for patterns in community structure associated with space and habitat, respectively.

We then tested whether species associated with warmer‐breeding habitats were moving into the region and species associated with colder habitats were moving out of the region. We modeled the relationship between the percent change in a species' observed occupancy and its species temperature index (STI), the long‐term average temperature across the species' breeding range (Devictor et al., 2008). The two species with the lowest and highest STI, respectively, Red Knot and Stilt Sandpiper, were excluded from this analysis as they were not observed during the early survey effort. To calculate STI, we used the breeding season occurrence maps available from Birdlife International to define the North American breeding range of each species (BirdLife International and Handbook of the Birds of the World, 2020). We calculated the mean June temperature (1970–2000) for each species' breeding range from the WorldClim 2.1 dataset, which has a 30 arc second (~1 km^2^) resolution (Fick & Hijmans, 2017). We chose to use this long‐term average climate to match with the spatial temporal scale that seems relevant to the species range data described above, which is necessarily coarse. We used the mean June temperature because shorebirds arrive in the region, initiate their nests, and begin incubation in June, and temperature influences these behaviors (Meltofte et al., 2007). We clipped the mean June temperature grid to our breeding range polygons, and calculated a mean value for June temperature across the whole of each species' breeding range. Finally, we used a linear model to test if there was a significant relationship (*p* < .5) between the percent change in a species' occupancy and its STI. The data used for these analyses are published in Anderson et al. (2022).

All analyses were done using R 4.2.1 (R Core Team, 2022) and RStudio 2022.7.2.576 (RStudio Team, 2022) and the tidyverse package 1.3.2 (Wickham et al., 2019).

## 3 RESULTS

**FIGURE 4:**
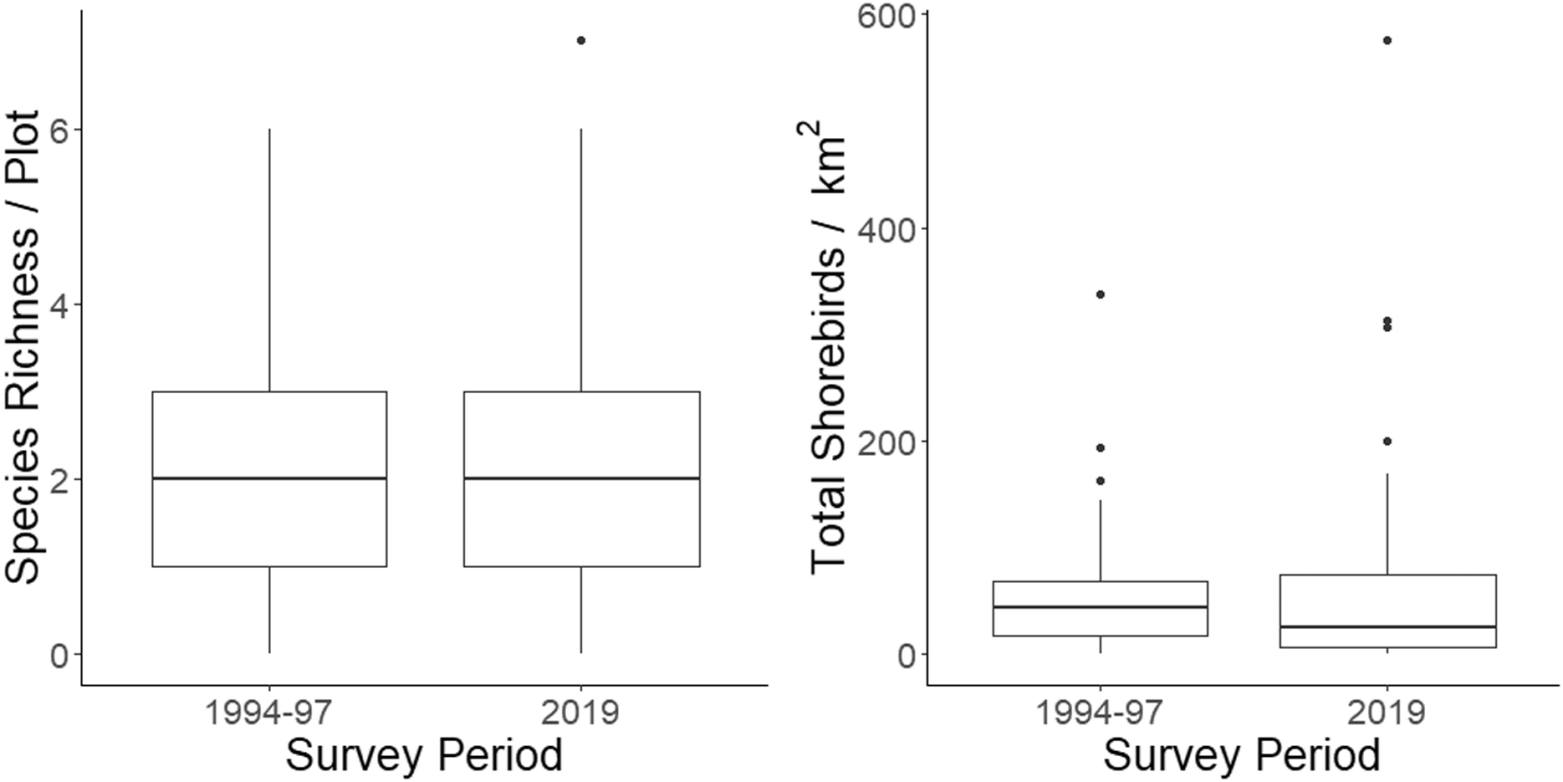
Species richness (right) and density of breeding shorebirds per km^2^ (left) observed in the two time periods of our study, for both regions combined.

There was considerable variability in the occupancy, richness, and density of breeding shorebirds between plots, therefore, there was no significant difference between 1994–1097 and 2019 (Table 1). The occupancy of breeding shorebirds per plot (all species combined) in the two study regions was 79% in 1994–1997 to 81% in 2019. The median (±SD) species richness per plot was 2 (±1.42) species per plot in 1994–1997 and 2 (±1.64) species per plot in 2019 (Figure 4). The median density (±SD) of breeding shorebirds was 44 (±59) birds/km^2^ in 1994–1997 and 25 (±94) birds/km^2^ in 2019 (Figure 4).

**TABLE 1:** Generalized linear model results for change in occupancy, richness, and density of breeding shorebirds (all species) from 1994–1997 to 2019 in Foxe the Rasmussen Lowlands and Foxe Basin (*n* = 64). The models included time period as the main predictor of interest, region to control for any regional effects, and habitat to control for any habitat effects (of upland and lowland habitat). The occupancy model used a binomial distribution, and the species richness and density models used negative binomial distributions. The intercept represents the parameter estimate for reference categories (1994–97, Foxe Basin, lowland). The estimate for the parameters in brackets (2019, Rasmussen, upland) represents the difference for that category and the reference level.

Model	Coefficient	Estimate	SE	Z	*p*
*Occupancy*	Intercept	3.83	0.43	8.76	>.01
	Time Period (2019)	0.10	0.46	0.22	.82
	Region (Rasmussen)	−1.03	0.47	−2.21	.02
	Habitat (Upland)	−0.54	0.56	−0.98	.33
*Richness*	Intercept	2.52	0.12	21.34	>.01
	Time Period (2019)	0.15	0.14	1.12	.26
	Region (Rasmussen)	−0.14	0.14	−1.03	.31
	Habitat (Upland)	−0.30	0.20	−1.52	.13
*Density*	Intercept	4.38	0.17	25.23	>.01
	Time Period (2019)	0.11	0.20	0.52	.42
	Region (Rasmussen)	−0.75	0.21	−3.62	.29
	Habitat (Upland)	−1.15	0.29	‐	>.01

**FIGURE 5:**
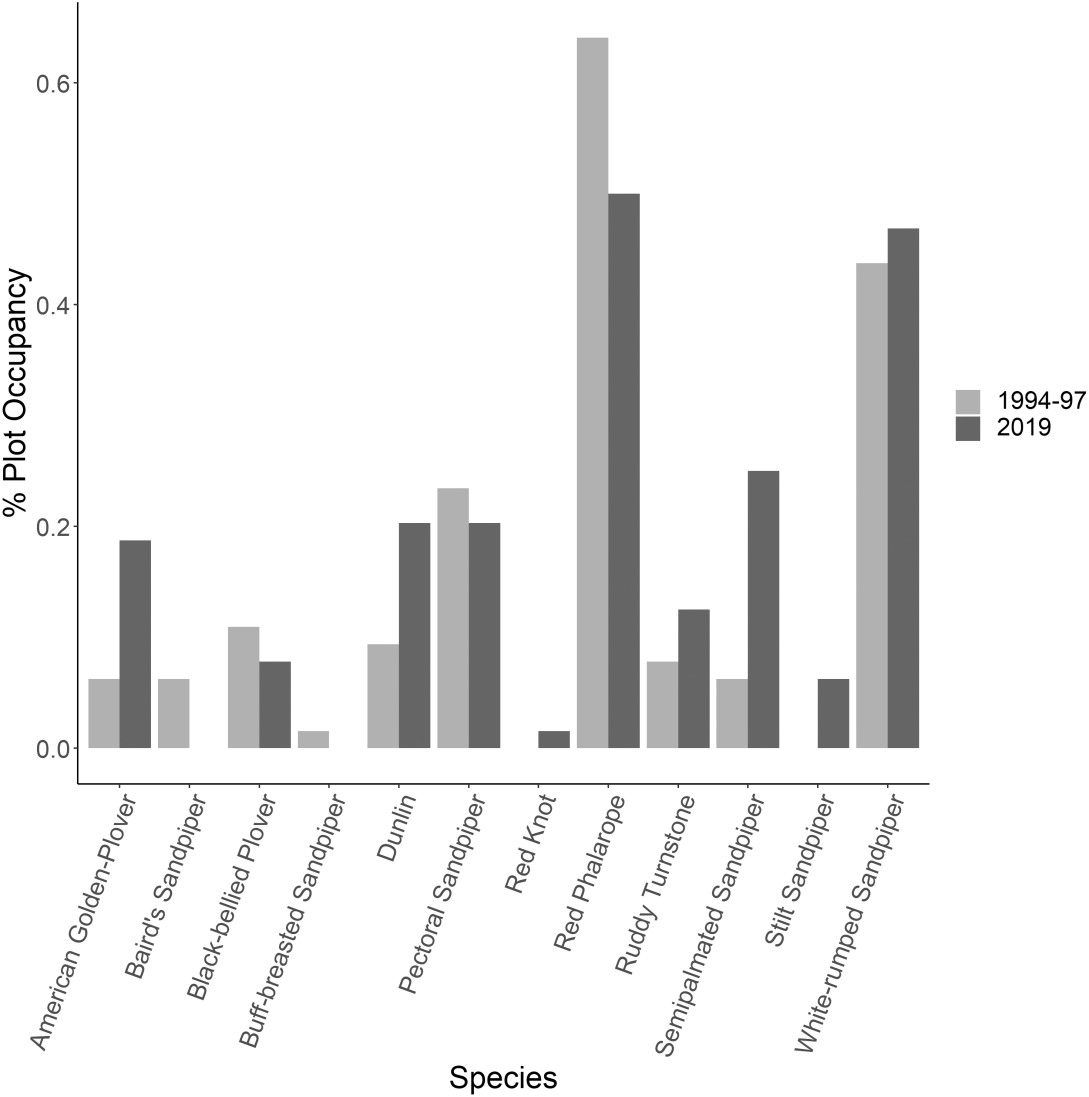
Change in individual species occupancy in plots surveyed in 1994–1997 and 2019 (*n* = 64) in both study regions combined.

**FIGURE 6:**
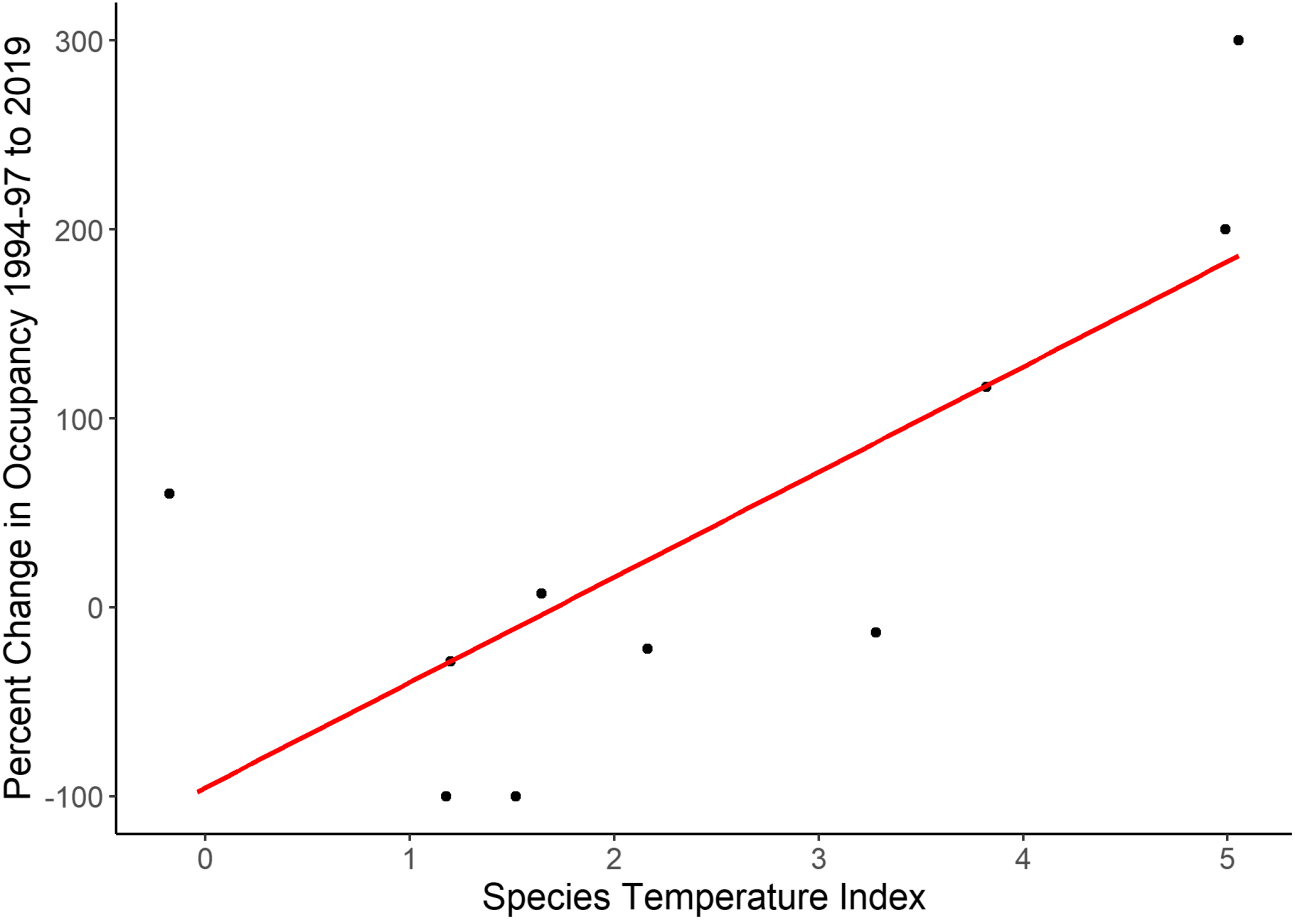
Linear model of the relationship between the percent change in occupancy of shorebird species from 1994–1997 to 2019 and their species temperature index. Intercept = −95.49, slope = 55.72, *p* = .01, and adjusted R^2^ = 0.51. The species temperature index is the mean June temperature from 1970 to 2000 across the species range. See Table 2 for a legend of four‐letter bird species codes.

Changes in occupancy were highly variable between species (Figure 5): occupancy by Baird's Sandpiper, Buff‐breasted Sandpiper, Black‐bellied Plover, Pectoral Sandpiper, and Red Phalarope declined (Table 2); occupancy by Ruddy Turnstone and White‐rumped Sandpiper increased moderately; and occupancy by Dunlin, American Golden Plover, and Semipalmated Sandpiper increased considerably. Interestingly, these same three species that increased substantially are the three species with the highest STI (Table 2). As predicted, there was a significant, positive relationship between the change in a species' occupancy and its STI (Figure 6; intercept = −95.49, slope = 55.72, SE = 17.32, *p* = .01, adjusted R^2^ = 0.51). STI ranged from −1.3°C for Red Knot to 5.3°C for Stilt Sandpiper (Table 2).

**TABLE 2:** Species temperature index and change in occupancy between 1994–1997 and 2019 for eight shorebird species

Species	Four‐letter species code	Species temperature index (°C)	% occupancy 1994–97	% occupancy 2019	% Change in occupancy
Red Knot	REKN	−1.3	0.0	1.6	Inf
Ruddy Turnstone	RUTU	−0.2	7.8	12.5	60
Baird's Sandpiper	BASA	1.2	6.2	0.0	−100
Black‐bellied Plover	BBPL	1.2	10.9	7.8	−29
Buff‐breasted Sandpiper	BBSA	1.5	1.6	0.0	−100
White‐rumped Sandpiper	WRSA	1.6	43.8	46.9	7
Red Phalarope	REPH	2.2	64.1	50.0	−22
Pectoral Sandpiper	PESA	3.3	23.4	20.3	−13
Dunlin	DUNL	3.8	9.4	20.3	117
American Golden Plover	AMGP	5.0	6.3	18.8	200
Semipalmated Sandpiper	SESA	5.1	6.3	25.0	300
Stilt Sandpiper	STSA	5.3	0.0	6.3	Inf

## 4 DISCUSSION

Our results indicate shifting distributions for shorebirds in Arctic Canada at a large spatial scale over a period of 25 years. We found that occupancy varied widely across species, some increasing and some decreasing, despite the negative population trends observed for these species based on migratory data from southern Canada and the United States (Bart & Johnston, 2012; Smith et al., 2020, 2023). The changes in occupancy that we observed were positively related to STI. The increases in occupancy by Dunlin, American Golden Plover, and Semipalmated Sandpiper, the warmer‐breeding species, indicate that these species may be moving into these regions. Most of the colder‐breeding species, namely Baird's Sandpiper, Buff‐breasted Sandpiper, Black‐bellied Plover, Pectoral Sandpiper, and Red Phalarope, were observed less frequently, potentially as their ranges shifted northward.

Species distributions are shaped by complex interactions among abiotic conditions, biotic interactions, dispersal capabilities, and historical events, operating at different intensities at different spatial scales (Gaston, 2003). Climate is widely recognized as one of the most common, influential drivers of species distribution, through both direct and indirect effects (Grinnell, 1917; Root, 1988). All of the species considered here have breeding ranges centered at Arctic latitudes, but their breeding distributions nevertheless vary widely in terms of climate. Arctic‐breeding shorebirds arrive to breed as the snow recedes in May and June, and their fledged young must depart before snow returns in August and September; temperature and weather during this brief window can have a profound effect on reproductive success (Meltofte et al., 2007). This may be through direct effects on incubation and chick survival during extreme events, or through indirect effects on invertebrate prey availability, the timing of snow, and the vegetation community (Kwon et al., 2019; Pearce‐Higgins et al., 2010; Swift et al., 2017; Tulp & Schekkerman, 2008). Climate change during the non‐breeding season also plays a role, for example, through inundation of coastal habitats (Galbraith et al., 2002). Decreased survival during the non‐breeding season could influence distributions on the breeding grounds if there is strong migratory connectivity between breeding and wintering sites (Iwamura et al., 2013).

Climate change could also have indirect effects on shorebird distributions through its effect on biotic interactions (Blois et al., 2013). Climatic shifts appear to lengthen lemming population cycles in the Arctic, and decrease their maximum population densities (Gilg et al., 2012). This is likely to affect shorebird distributions, as the presence of lemmings provides alternative prey for Arctic foxes (Gilg & Yoccoz, 2010; Léandri‐Breton & Bêty, 2020), reducing predation risk in shorebird nests. Climate‐related northward shifts of nest predator distributions could also increase nest loss through predation at higher latitudes, nest predation risk having been shown to decrease with latitude (McKinnon et al., 2010).

The status and distribution of shorebirds is undoubtedly influenced by non‐climatic factors as well. The densities of Dunlin, Pectoral Sandpiper, Red Phalarope, Semipalmated Sandpiper, and White‐rumped Sandpiper are depressed in the vicinity of Snow Goose (*Anser caerulescens*) and Ross' Goose (*Anser rossi*) colonies, for which populations and colonies have increased dramatically in the past century, in large part due to increasing agricultural food subsidies in their overwintering areas (Flemming et al., 2019). In their migration and wintering habitats, shorebird survival has been negatively affected by processes including loss of coastal habitats to development (Fernández & Lank, 2008; Murray & Fuller, 2015) and unsustainable hunting of some species (Watts et al., 2015). Semipalmated Sandpipers have shifted their stopover habitats in response to increasing predation as raptor populations recover from critical lows, a dynamic that is likely affecting other shorebird species as well (Hope et al., 2020).

All studied species, including the four species showing increasing occupancy in our two study regions, are thought to be declining in total abundance based on surveys during migration at temperate latitudes in Canada and the United States (Smith et al., 2023). These declines average around 50% over 15 years and appear to be accelerating when compared to the previous 15 years. The mismatch between the trends we observed in the eastern Arctic and the overall population trends for Semipalmated Sandpiper, Ruddy Turnstone, Dunlin, and American Golden Plover suggests that our study regions may have been closer to the margins of these species' ranges 25 years ago, and that climate change has shifted their distributions such that our regions are now closer to the center of their ranges, where occupancy is assumed to be higher. For the species showing declines in occupancy, our data are likely reflecting a shift or contraction of their ranges toward the north as well as overall population declines. The inference of range shifts is stronger for the four species showing simultaneously increased occupancy in our region and overall population declines. It is interesting to note that we also observed a decline in the density of all breeding shorebirds in these two regions, although the result was not significant.

A similar study of shorebird population trends in Fennoscandia found that there was no relationship between breeding latitude and species population trends (Lindström et al., 2019). The authors discuss how the relationship between climate and latitude in this region is complicated by altitudinal west–east climate gradients. The area for which they calculated a species mean latitude may also have been too small to fully capture the range of conditions these species inhabit (14° of latitude vs. 25° of latitude in the current study). Using metrics such as STI which more closely reflect the conditions that species experience is likely more useful than assessing relationships between species population trends and latitude.

Given the numerous other factors and interactions influencing shorebird distributions, it is notable that we detected an apparent signal of climate change through our cross‐species analysis. As climate change in the Arctic is expected to be rapid and severe, environmental changes to shorebird‐breeding habitats may increasingly cause additional stress in these species. Changing distributions on the Arctic‐breeding grounds, including local increases in occupancy and density in some cases, indicate that suitable habitat continues to exist in the Arctic for some species. However, there are limits to these species' capacity to shift their ranges, especially for colder‐breeding species, not least of which is the geographic limit imposed by the Arctic Ocean. The shift toward a warmer‐breeding community of species suggests that, in addition to the pressures on shorebird species during the non‐breeding periods, we should also be concerned about declining breeding habitat availability for shorebird species whose current breeding ranges are centered on higher, colder latitudes.

## AUTHOR CONTRIBUTIONS

**Christine Anderson:** Conceptualization (equal); data curation (supporting); formal analysis (lead); methodology (equal); validation (lead); visualization (lead); writing – original draft (lead); writing – review and editing (lead). **Lenore Fahrig:** Conceptualization (equal); formal analysis (supporting); methodology (equal); supervision (supporting); writing – original draft (supporting); writing – review and editing (supporting). **Jennie Rausch:** Data curation (lead); funding acquisition (lead); writing – review and editing (supporting). **Jean‐Louis Martin:** Conceptualization (supporting); funding acquisition (supporting); writing – review and editing (supporting). **Tanguy Daufresne:** Conceptualization (supporting); funding acquisition (supporting); writing – review and editing (supporting). **Paul A. Smith:** Conceptualization (equal); data curation (supporting); formal analysis (supporting); funding acquisition (lead); methodology (equal); project administration (lead); resources (lead); supervision (lead); writing – original draft (supporting); writing – review and editing (supporting).

## CONFLICT OF INTEREST

The authors have no conflict of interest to declare.

## Data Availability

The data that support the findings of this study are openly available on Zenodo at https://doi.org/10.5281/zenodo.6653821 ‐ “Shorebird survey data from Foxe Basin and Rasmussen Lowlands, Nunavut ‐ 1994‐97 and 2019”.
